# Supplementary material for: Scribble, Lgl1, and myosin IIA interact with α-/β-catenin to maintain epithelial junction integrity
Source: Cell Adh Migr. 2023 Sep 24;17(1):1–23. doi: 10.1080/19336918.2023.2260645 (PMC10761038; doi:10.1080/19336918.2023.2260645)
Supplement: Supplemental Material [file KCAM_A_2260645_SM4971.zip › Figure_S1_S5_word_document_120723.docx]

1. **A549-tet-shLgl1**
2. **A549-tet-shScrib**

110kDa 210kDa 135kDa 102kDa

|  |  |
| --- | --- |
|  |  |
|  |  |
|  |  |
|  |  |
|  |  |
|  |  |
|  |  |
|  |  |
|  |  |
|  | 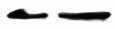 |
|  |  |

92kDa 48kDa


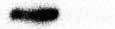
 **IB: anti Lgl1
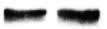
** **IB: anti Scrib
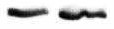
** **IB: anti E-cadherin
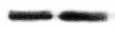
** **IB: anti α-catenin
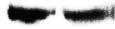
** **IB: anti β-catenin**

**IB: anti Actin**

210kDa 110kDa

135kDa 102kDa 92kDa 48kDa

|  |  |
| --- | --- |
|  |  |
|  |  |
|  |  |
|  |  |
|  |  |
|  |  |
|  |  |
|  |  |
|  |  |
|  | 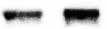 |
|  |  |


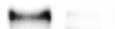
 **IB: anti Scrib
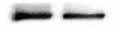
** **IB: anti Lgl1
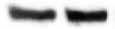
** **IB: anti E-cadherin
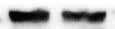
** **IB: anti α-catenin
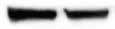
** **IB: anti β-catenin**

**IB: anti Actin**

135kDa 110kDa 210kDa

1. **D.**

210kDa


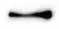

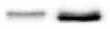

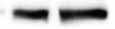

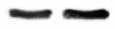

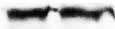

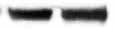

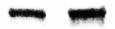

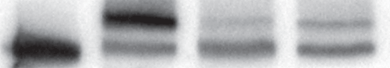

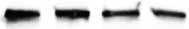

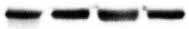

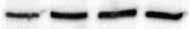

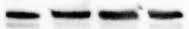

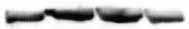


**IB: anti Lgl1**

210kDa

**IB: anti Scrib**

**IB: anti Scrib IB: anti GFP**

135kDa 102kDa 92kDa

48kDa

**Figure S1**

**Supplemental Figure S1:** The expression of Scrib, Lgl1, E-cadherin, and α- and β-catenin were analyzed in Lgl1- **(A)** and Scrib- **(B)** depleted cell lines, as well as in cells expressing Neon-Lgl1 proteins **(C)** or GFP-Scrib **(D)**. Actin served as a loading control. Black and green arrows in **C**, indicate endogenous and Neon fusion proteins, respectively. Molecular weights of the proteins are indicated.

**IB: anti E-cadherin IB: anti α-catenin IB: anti β-catenin IB: anti Actin**

110kDa

135kDa 102kDa 92kDa 48kDa

**IB: anti Lgl1**

**IB: anti E-cadherin IB: anti α-catenin IB: anti β-catenin IB: anti Actin**

**A.** **E-cadherin**

*p*<0.0001

| *ns* |  |
| --- | --- |
| *p*<0.0001 |  |
| *p*<0.0001 | |

*p*<0.0001


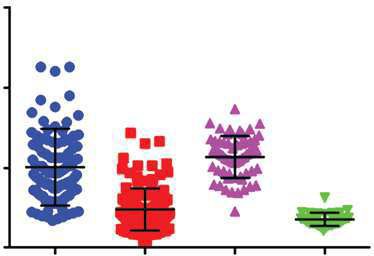
**30**

**Signal** **Intensity**

**(Junctional/Cytoplasmic)(A.U)**

**20**

**10**

**0**

## Scrib

*p*<0.0001

**B.**

| *ns* |  |
| --- | --- |
| *p*<0.0001 |  |
| *p*<0.0001 | |

*p*<0.0001


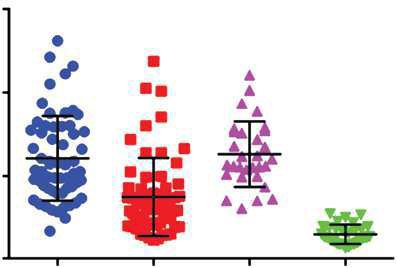
**30**

**Signal** **Intensity**

**(Junctional/Cytoplasmic)(A.U)**

**20**

**10**

**0**

**C.** **α-catenin D.**

*p*<0.0001


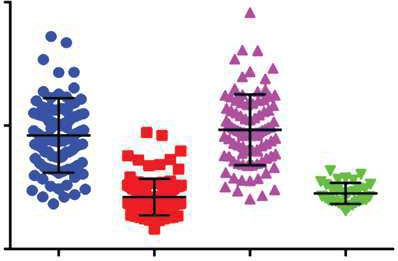


*p*<0.0001

*ns*

*p*<0.0001

*p*<0.0001

| *ns* |  |
| --- | --- |
| *p*<0.0001 |  |
| *p*<0.0001 | |

*p*<0.0001


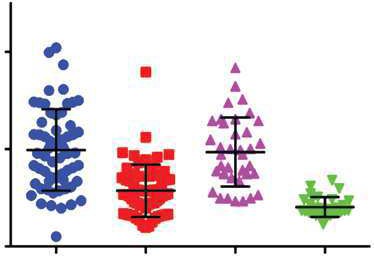
**20**

**Signal** **Intensity**

**(Junctional/Cytoplasmic)(A.U)**

**Signal** **Intensity**

**(Junctional/Cytoplasmic)(A.U)**

## β-catenin

*p*<0.0001

**20**

**10**

**10**

**0** **0**

**Figure S2**

**Supplemental Figure S2:** Dot-plot of signal intensity of junctional protein in comparison to cytoplas-

mic protein of E-cadherin **(A)**, Scrib **(B)**, α-catenin **(C)**, and β-catenin **(D)** in the indicated Lgl1 cell lines. Values are the mean ± SD from three independent experiments subjected to ANOVA, with a post hoc test. *ns*: not signiﬁcant.

## E-cadherin

*ns*


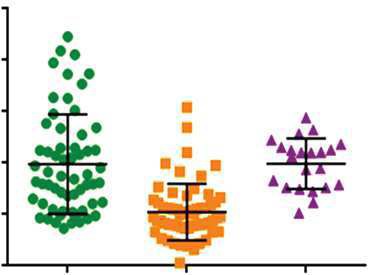


*p*<0.0001

*p*<0.0001

**25**

**Signal** **Intensity**

**(Junctional/Cytoplasmic)(A.U)**

## Lgl1

*ns*


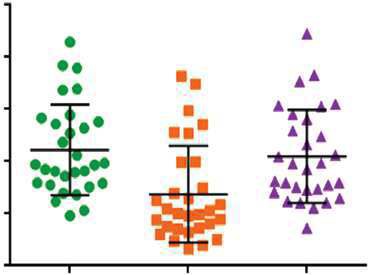


*p*=0.003

*p*=0.0006

**5**

**Signal** **Intensity**

**(Junctional/Cytoplasmic)(A.U)**

**20** **4**

**15** **3**

**10** **2**

**5** **1**

**0**

**Ctrl** **Scrib^KD^** **Scrib-Rescue**

**0**

**Ctrl** **ScribKD** **Scrib-Rescue**

## α-catenin

*ns*


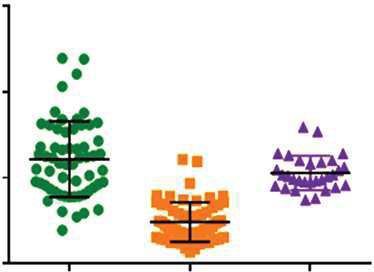


*p*<0.0001

*p*<0.0001

**30**

**Signal** **Intensity**

**(Junctional/Cytoplasmic)(A.U)**

## β-catenin

**20**


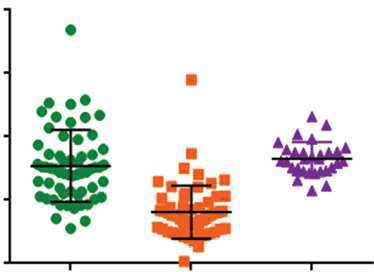


*ns*

*p*<0.0001

*p*<0.0001

**Signal** **Intensity**

**(Junctional/Cytoplasmic)(A.U)**

**15**

**20**

**10**

**10**

**5**

**0**

**Ctrl** **ScribKD** **Scrib-Rescue**

**0**

**Ctrl** **ScribKD** **Scrib-Rescue**

**Figure S3**

**Supplemental Figure S3:** Dot-plot of signal intensity of junctional protein in comparison to

cytoplasmic protein of E-cadherin **(A)**, Lgl1 **(B)**, α-catenin **(C)**, and β-catenin **(D)** in the indicated Scrib cell lines. Values are the mean ± SD from three independent experiments subjected to ANOVA, with a post hoc test. *ns*: not signiﬁcant.

| **A.** |  | | | |
| --- | --- | --- | --- | --- |
| 210kDa | 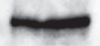 | 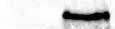 | | **IB: anti Scrib** |
| 135kDa | 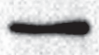 | 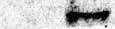 | | **IB: anti E-cadherin** |
| **B.** |  | **MBP-Lgl1** | |  |
| **Input** | | | **PD:** **GST** | |

120kDa

|  |  |  |
| --- | --- | --- |
|  |  |  |
|  |  |  |
|  |  |  |

30kDa 140kDa


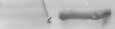
 **GST-β-catenin**


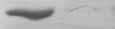
 **GST-only**

|  | 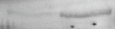 |  |
| --- | --- | --- |
|  |  |  |
|  | 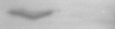 |  |
|  |  |  |
|  | 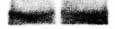 | 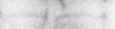 |
|  |  |  |

**IB:** **MBP-Lgl1**

**Figure S4**

**Supplemental Figure S4: (A)** A549 cell extracts were subjected to co-IP assay

using Scrib antibody. The immunoprecipitated proteins were analyzed by IB with antibodies against Scrib and E-cadherin. IgG was used as negative control. **(B)** MBP-Lgl1 and GST only or GST-β-catenin were subjected to PD assay. MBP-Lgl1 was analyzed by IB with antibody against MBP-tag, and GST proteins were analyzed by Ponceau S staining. Molecular weights of the proteins are indicated.

# A.

## Days with OHT 0 3 6

|  |  |
| --- | --- |
|  |  |
|  |  |
|  |  |
|  |  |
|  |  |

210kDa
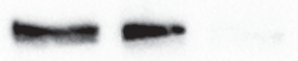
 110kDa
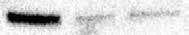
 48kDa
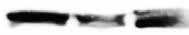


# B.

## Anti Scrib Anti Lgl1 Anti Actin

210kDa 110kDa 135kDa

|  | 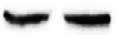 |
| --- | --- |
|  |  |
|  | 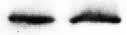 |
|  |  |
|  | 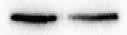 |
|  |  |
|  | 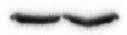 |
|  |  |

48kDa

**Figure S5**

**Supplemental Figure S5: (A)** HMLE-Twist-ER cells were

induced by 4-hydroxytamoxifen (OHT) for the indicated time points, and cell lysates were analyzed by IB with antibodies against Scrib and Lgl1. Actin served as a loading control. **(B)** A549 cells were incubated with TGFβ for 16 h, and cell lysates were analyzed by IB with antibodies against Scrib, E-cadherin, and Lgl1. Actin served as a loading control. Molecular weights of the proteins are

**IB: Anti Scrib IB: Anti Lgl1**

**IB: Anti E-cadherin IB: Anti Actin**
